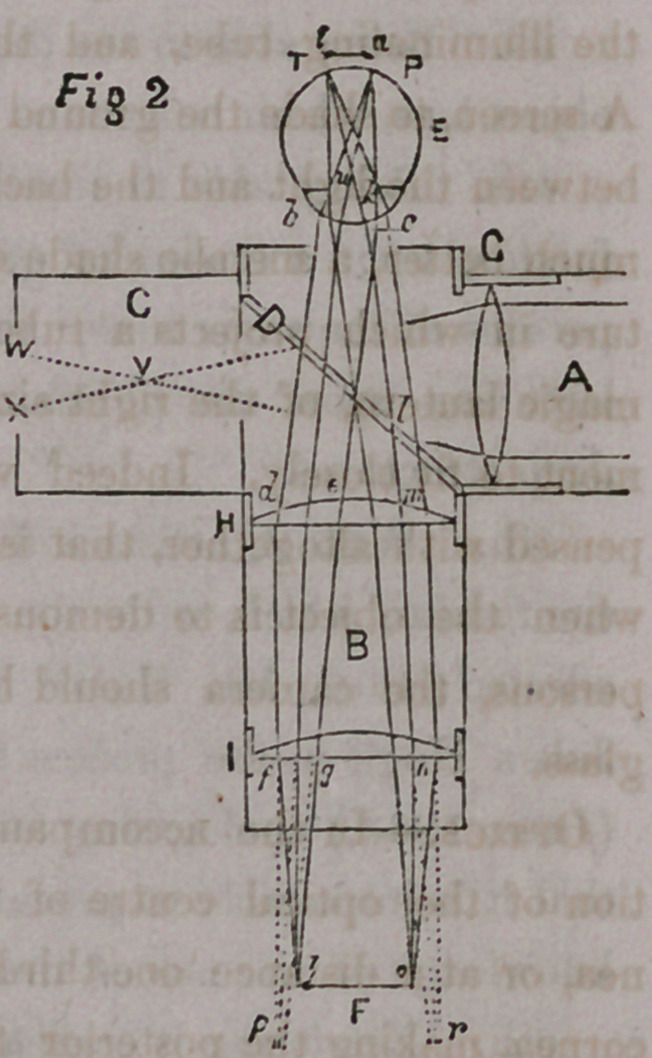# On a New Instrument for Photographing the Fundus Oculi

**Published:** 1864-05

**Authors:** A. M. Rosebrugh

**Affiliations:** Toronto


BUFFALO

### Medical and Surgical Journal.

VOL. III. MAY, 1864. No. 10.

ART. I.—*On a New Instrument for Photographing the Fundus Oculi.* By A. M. Rosebrugh, M. D., *Toronto.*

It is well known that under ordinary circumstances, the pupil of the eye appears quite black, and all parts behind it are perfectly insensible. This was formerly thought to depend upon the total absorption, by the choroid, of all the rays of light that fall upon the fundus of the eye. But since the invention of the Ophthalmoscope it has been very satisfactorily demonstrated that the phenomenon just referred to depends solely upon the refraction the rays of light undergo in passing through the ocular media, and that a sufficient number of these rays are reflected from the interior of the eye to be visible to the eye of an observer placed in a proper position to receive them.

"When a perfectly formed eye is exactly accommodated for a luminous object, the diverging rays from this, incident upon the eye, are refracted by the ocular media in such a manner that they unite at a point in the surface of the retina which is the image of that object. The retina, in consequence of its transparency, transmits much of this light to the choroid, by which most of it is absorbed; but many of these rays are reflected in the same direction in which they entered the eye, and return to the object whence they started."*

These reflected returning rays would be visible to the eye of an observer placed in line with the light and the eye under examination, but without some special contrivance for the purpose, this position is an impossible one as, if the experimenter places his eye beyond the light his eye is

* Hulke, treatise on the Ophthalmoscope.

dazzled, and if it is placed between the light and the eye under examination, the illuminating rays are intercepted. This is effected in the ophthalmoscope by substituting reflected for direct light. In Liebreich's small ophthalmoscope, the one now in general use by ophthalmoscopists, the light of a lamp is reflected into the eye from the surface of a small concave mirror, which is pierced by a central sight hole, through which the observer looks in the direction of the eye under examination, and is thus enabled to see the light reflected from its illuminated fundus.

We saw just now, that the light reflected from the eye, returns to the luminous object from which it emanates; this being the case these returning rays must be convergent, and as a normal eye is incapable of bringing convergent rays of light to a focus on its retina, the observer looking through the sight hole of the mirror, sees only the pinkish red color of the choroid, without (except in hypermebropic eyes) being able to distinguish any of the details of the illuminated fundus. Before these rays can be brought to a focus on the surface of the retina of the observer they must be made parallel or slightly divergent. This is effected by placing a convex lens over the sight hole of the mirror. This is called the *direct* method of examination in contra distinction to the indirect method about to be described where the observer sees the *inverted aerial* image of the fundus oculi.

In this indirect method, the mirror is held twelve or fourteen inches from the patient's eye and a double convex lens of about two inch focus is placed from one to two inches from the eye, which has the effect of bringing the reflected light from the illuminated fundus to a focus nearly two inches in front of the lens, or about three inches in front of the eye under examination, where the observer looking through the sight hole of the mirror sees an inverted aërial image of the posterior internal surface of the eye.

In the modification of this instrument which I have recently invented, this aërial image of the eye ground is received upon a screen of ground glass which can be seen by a number of persons at the same time; and by substituting a prepared photographic plate for the ground glass, photographs are taken, showing the details of the deep structures of the living eye.

This instrument is constructed upon the principle that plate glass has the property of partly reflecting and partly refracting rays of light that are incident upon its surfaces.*

* This is also the principle upon which Prof. Pepper produces the appearance of the *ghost* upon the stage.

CONSTRUCTION.

*The Tubes.*—The instrument consists of two brass tubes, (A and B, fig. 1,) 1½ inches in diameter, being respectively 4 and 2½ inches in length. The longer tube B moves freely in a brass collar fitted to the aperture of a small camera K, and the shorter tube A is turned toward the source of light.

A tube C of the same width, 1½ inches in length, is joined to the side of the outer extremity of the tube B opposite to and in a line with tube A. The outer extremity of the tube B extends ¼ of an inch beyond its juncture with the tubes A and C, and is terminated by a thin brass diaphragm, having a central circular aperture of ⅜ of an inch in diameter.

At the juncture of the tube A with B there is a circular aperture of one inch diameter, and between C and B an aperture of ½ inch diameter, affording a communication between A and C through B.

*The Plate Glass.*—At the juncture of the tubes, there is placed an elliptical piece of highly polished thin plate glass, with parallel surfaces, which is inclined at such an angle to the tubes that a ray of light falling upon it through the centre of the tube A from the direction M Q will be reflected at right angles to its original direction and in the same plane with the centre of the tube B, which will be through the centre of the aperture in the diaphragm. A portion of the ray will be refracted by the plate glass, and pass through the tube C parallel to its original direction.

*The Lenses.*—At the inner extremity of the illuminating tube A, and as close as possible to its juncture with the camera tube B, a double convex lens G is placed 1½ inches in diameter, and having a focal distance of 2½ inches. In the corresponding position of the tube B, or close to the plate glass reflector, the lens H is placed, convexo-plane of 5 inch focal distance; 1⅝ inches from this is another lens, I, also convexo-plane, and of 5 inch principal focal distance, and having the same diameter, viz, 1¼ inch.*

*The Camera.*—The Camera consists of a mahogany box three inches square and seven inches high, having (to secure steadiness) a base six inches

*I have ascertained that a single lens of 2½ inch focus answers quite as well as the two (H and I) of 5 inch each.

square. At the aperture in the centre of the anterior side there is a brass collar fitted, through which slides the tube B containing the lenses. At the opposite side of the camera is a central aperture 2½ inches square, behind which is a slide with a piece of ground glass 2½ inches square. This slide moves in grooves for the purpose, and can be removed to make way for a slide containing a sensitized plate also about 2½ inches square. The whole is contained in a case about 8 inches in height, which serves the double purpose of supporting the instrument when in use, and holding it afterwards.

PHOTOGRAPHING.—As yet I have not attempted a photograph of the retina of the human eye, but have confined my experiments to the lower animals, and have employed solar light only in order to shorten the time as much as possible; but I do not doubt that diffused light, particularly that reflected from a bright cloud, would with a longer "exposure" answer very well. In using the instrument for this purpose, a tripod, or what answers quite as well, a table of the ordinary height is placed near a window where the light of the sun will fall upon it.

It is well to have the shutters closed, and a beam of solar light admitted of the size of the illuminating tube; but this is not absolutely essential if precautions be taken to prevent diffused light entering the camera, and the ground glass be shaded while examining the image on its surface.

*Position of the Instrument.*—The camera must be turned at right angles to the source of light, and the tube A, or illuminating tube, turned so that the light will fall full into the tube, and be incident upon the whole of the lens G.

If the camera and tube be now in proper position, a cone of light will issue from the end of the camera tube through the centre of the aperture in the diaphragm, which is the condensed light fiom the lens G reflected from the plate glass D. This cone forms a focus about one-half inch outside the diaphragm, which can be seen by holding a thin piece of white paper near the diaphragm. If it be a cat, or rabbit, that is to be experimented upon, it is well to have it secured in a box of the right size, with the head projecting through an aperture for the purpose.

In photographing the eye of a cat I found it necessary to put it under the influence of chloroform, but the image of the optic nerve, vessels, etc., upon the ground glass is so very bright and clear that I do not doubt, if the most sensitive process be adopted, the impression could be taken instantaneously, thus rendering anæsthesia unnecessary.

*Position of Eye.*—In either case the eye must be brought to the proper position, and the eyelids held apart by an assistant. If it be the eye of a patient to be photographed, the instrument must be mounted upon its case, which will, for most persons, give it the right height. The patient being seated upon a chair as close as possible to the table, should lean forward toward the camera, and bring his eye as near as possible to the aperture in the diaphragm, the brow can rest lightly against the end of the tube, and by bringing the elbow upon the table he can, with the palms of his hands, extemporize a very good rest for his chin.

The pupil of the eye to be photographed must have been previously dilated with atropine.

*Process.*—If the instrument be now in its proper position, and the light from the plate glass enter the dilated pupil, the fundus of the eye will be brilliantly illuminated, and its reflection will pass out of the eye and through the plate glass and lenses, and form an inverted image upon the ground glass at the back of the camera, where the observer in the rear will see the optic nerve entrance, distribution of the arteries and veins, etc., beautifully depicted, but magnified about four diameters.

If the details of the image be not perfectly defined the camera tube must be moved backwards or forwards until the proper focus be obtained. This image can be seen by the observer again very much magnified by placing to his eye a lens of say six inch focal length, and bringing his eye with the lens to within six inches of the ground glass; but the image will be seen even better by moving the ground glass to one side; the observer will then see the *aerial* image of the reflection from the eye, which will occupy the same position as the ground glass previously occupied. The slide containing the ground glass can now be removed and a slide substituted containing a glass plate "prepared" by the ordinary collodion process. An "exposure" of about five seconds is sufficient. If the "developing" prove that a good "negative" has been obtained, it must be "fixed" and used for printing the photographs; if not, other plates should be employed until a more satisfactory result be obtained.

AS AN OPHTHALMOSCOPE.

*The position of the Instrument when the light is supplied by a lamp.*—A the camera, B camera tube, C illuminating tube, D diaphragm with central aperture, E slide with ground glass, F glass chimney of lamp, G brass tube which acts as a shade, and from which projects H, a brass collar opposite the flame of the lamp, and to which is adapted the illuminating tube C of the instrument; I, upright of the lamp-stand, J eye-piece to be adapted to the inner extremity of the camera tube B; when this is used, the camera can be dispensed with.

In using this instrument as an ophthalmoscope, that is, for examining the interior of the eye, artificial light should be employed. That from a kerosene oil lamp answers very well, but the best light for ophthalmoscopic purposes is from the gas argand-burner, and the most convenient is the movable table lamp supplied with gas through a flexible tube. The evening is the best time for making these examinations; if in the day time, the room must be darkened, and the instrument be placed in the same position in regard to the light as when solar light is used, by the flame of the lamp should be brought within two or three inches of the entrance of the illuminating tube, and the two must be on the same horizontal line.—A screen, to shade the ground glass and the observer's eye, should be placed between the light and the back of the camera, or, what I have found to be much better, a metalic shade can be placed around the lamp, from an aperture in which projects a tube or collar, somewhat resembling that of a magic lantern, of the right size to allow the illuminating tube of the instrument to fit closely. Indeed with this apparatus the camera can be dispensed with altogether, that is in making examinations of the eye simply; when the object is to demonstrate the fundus of the eye to a number of persons, the camera should be used both with and without the ground glass.

Optics.—In the accompanying diagrams I have made the mean position of the optical centre of the eye at the centre of curvature of the cornea, or at a distance one-third of the distance of the diameter from the cornea, making the posterior focal distance of the eye about two thirds of an inch. I have also represented the eyes as "homogenous bodies, possessed of a single condensing refracting surface, which is regarded as the "optical equivalent of the various surfaces in a real eye,

"By giving such hypothetical eyes a higher index of refraction than that "of the media of any real eye, we may preserve the proportion between "the distance of the cornea and the retina from the optical centre almost "unchanged, while substituting an equivalent for a real eye, which may be "assumed to be quite accurate in so far as concerns any optical conclusions "with which we have to do."—*Dr. George Rainy on the Theory of Ophthalmoscope.*

*Illumination.*—Let M Q (fig. 1) represent parallel rays of solar light incident upon the double convex lens G, at the points N R they are refracted and emerge from the lens convergingly towards a focus V in the tube C, but at O and S they are intercepted by the plate glass D, a portion of the rays are reflected by its polished surface in the direction E, and rays not reflected or absorbed are transmitted and pass to form a focus at V, the principal focal distance of the lens G, and again diverge in the direction W X.

The rays reflected from the surface of the plate glass form a focus at U (which is also the focal centre of the eye E) at the same distance in front of the plate glass D, as V is behind it, these rays at U again diverge and illuminate a portion of the fundus at T P.

*Reflection.*—Let E (fig. 2) represent the same eye, illuminated as just described, D the plate glass, and H I the lenses in the camera tube. Rays from any portion of the illuminated fundus as *a*, are reflected from the fundus and emerge from the cornea at *b c*, the width of the dilated pupil, and proceed to the plate glass D (parallel rays of light emerging from an eye having its accommodation paralyzed are parallel or very nearly so) where some of its rays will be reflected through the lens G in the direction of the source of illumination, but other rays proceed to *d*, *e*, where they are incident on the lens H by which they are refracted, and they would proceed to a focus at the principal focal distance of the lens H, viz: at 5 inches, but they are again intercepted at *f*, *g*, by the lens I, which refracts them to an earlier focus at *h*. In the same way rays from *i*, on E's retina, proceed from the cornea parallel to the axis *i*, *k*, *m*, and are also refracted by the lens H and I, and are brought to a focus at *o*. In like manner all points intermediate between *i* and *a*, on E's retina, are reflected from the fundus and refracted by the lenses forming an inverted image of *i*, *a*, at *o*, *h*, which is received upon the ground glass placed at F.

Fig 2

Application—Advantages.—The advantages I claim for this instrument are:—

1st. The simplicity of its construction, taking into consideration its twofold purpose, viz: as an ophthalmoscope, and as a photographing instrument. My friend, Dr. Noyes, of the New York Eye Infirmary, constructed an instrument for photographing the fundus oculi, and which was I believe to a considerable extent successful, but its construction was too complicated and the instrument too expensive to be generally adopted. Dr. Noyes' instrument is constructed somewhat upon the principle of the binocular microscope. Any good optician can construct this instrument. The one I exhibited to the Institute was made by Charles Potter, of King street, Toronto.

2d. The limited experience necessary in order to use it successfully; the ordinary ophthalmoscope requiring months of practice before it can be used satisfactorily.

3d. Being able to see the aerial image free from reflections from the object lens, which reflections are serious obstacles to beginners.

4th. Being able to receive the image, either of a healthy or diseased fundus, upon a screen of ground glass which can be seen by a number of persons at the same time, and could be taken advantage of by gentlemen lecturing upon the physiology of the eye, or upon the pathology of its deep structures.

5th. With it, artists will be enabled to make colored representations of the fundus, which, with the instruments now in use, has never yet been effected; thus, Mr. Hulke in his Treatise on the Ophthalmoscope, and Jabez Hogg in the preface to his "Manual of Ophthalmoscopic Surgery," (June, 1863,) apologizing for defects in their colored representations, state that it is impossible to procure the services of artists having the requisite knowledge of the use of the ophthalmoscope.

6th. Rendering it comparatively easy to photograph the reflection from the posterior internal surface of the eye.

I cannot conclude without expressing the hope that this instrument will contribute something towards awakening more of an interest in ophthalmoscopic science, as the ophthalmoscope is undoubtedly as essential in investigating diseases of the eye, as the stethescope in diagnosing affections of the heart and lungs; and I trust its use will aid in banishing from ophthalmic nomenclature the indefinite term of amaurosis, where, as Walther observed, "the patient and physician are equally blind."

## Figures and Tables

**Fig. 1. f1:**
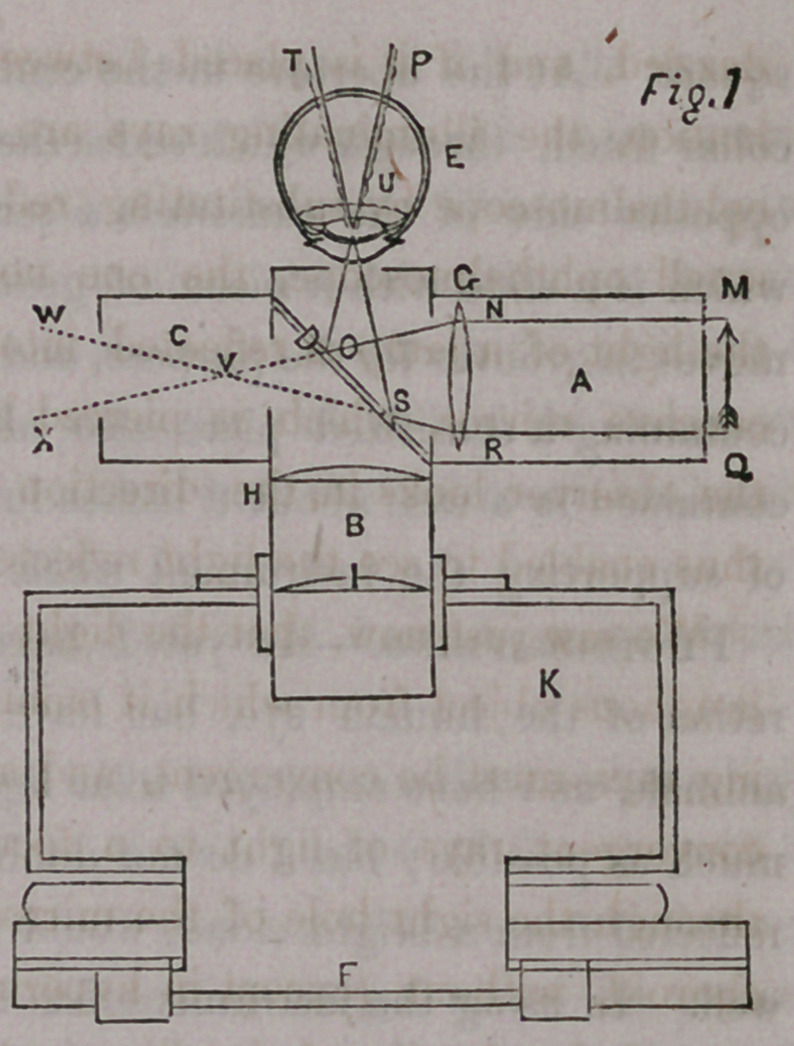


**Figure f2:**
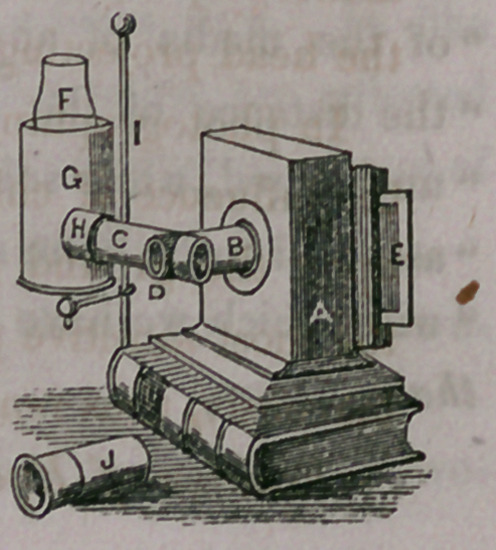


**Fig. 2. f3:**